# Simplified production and concentration of HIV-1-based lentiviral vectors using HYPERFlask vessels and anion exchange membrane chromatography

**DOI:** 10.1186/1472-6750-9-10

**Published:** 2009-02-16

**Authors:** Robert H Kutner, Sharon Puthli, Michael P Marino, Jakob Reiser

**Affiliations:** 1 Gene Therapy Program, Vector Core, Louisiana State University Health Sciences Center, New Orleans, LA, USA; 2 Division of Cellular and Gene Therapies, Office of Cellular, Tissue and Gene Therapies, US Food and Drug Administration, Center for Biologics Evaluation and Research, Bethesda, MD, USA

## Abstract

**Background:**

During the past twelve years, lentiviral (LV) vectors have emerged as valuable tools for transgene delivery because of their ability to transduce nondividing cells and their capacity to sustain long-term transgene expression in target cells *in vitro *and *in vivo*. However, despite significant progress, the production and concentration of high-titer, high-quality LV vector stocks is still cumbersome and costly.

**Methods:**

Here we present a simplified protocol for LV vector production on a laboratory scale using HYPERFlask vessels. HYPERFlask vessels are high-yield, high-performance flasks that utilize a multilayered gas permeable growth surface for efficient gas exchange, allowing convenient production of high-titer LV vectors. For subsequent concentration of LV vector stocks produced in this way, we describe a facile protocol involving Mustang Q anion exchange membrane chromatography.

**Results:**

Our results show that unconcentrated LV vector stocks with titers in excess of 10^8 ^transduction units (TU) per ml were obtained using HYPERFlasks and that these titers were higher than those produced in parallel using regular 150-cm^2 ^tissue culture dishes. We also show that up to 500 ml of an unconcentrated LV vector stock prepared using a HYPERFlask vessel could be concentrated using a single Mustang Q Acrodisc with a membrane volume of 0.18 ml. Up to 5.3 × 10^10 ^TU were recovered from a single HYPERFlask vessel.

**Conclusion:**

The protocol described here is easy to implement and should facilitate high-titer LV vector production for preclinical studies in animal models without the need for multiple tissue culture dishes and ultracentrifugation-based concentration protocols.

## Background

LV vectors provide powerful tools for transgene delivery into dividing as well as nondividing cells and for long-term transgene expression in target cells *in vitro *and *in vivo*. Currently, the use of LV vectors is commonplace and applications in the fields of neuroscience, hematology, developmental biology, stem cell biology and transgenesis have emerged [1]. LV vectors are also being pursued in a number of clinical trials (see ). Despite significant progress, the production and concentration of high-titer, high-quality LV vectors for preclinical studies in animal models is still cumbersome and costly [2].

The production of LV vectors is typically carried out using transient transfection approaches involving tissue culture dishes or flasks [3], cell factories [4-6], or stirred-tank bioreactors [7]. These protocols are cumbersome to scale up (dishes, flasks) or technically challenging (cell factories, bioreactors), preventing their routine use in a standard laboratory setting.

Typical LV vector titers involving the vesicular stomatitis virus (VSV) G glycoprotein range from 10^6 ^to 10^8 ^TU/ml [8]. Higher titers can be achieved by physical concentration [2], including ultracentrifugation [3,9-11], or filtration approaches such as ultrafiltration [3,10,12-14], and diafiltration [4,6]. Vector production for large-scale *in vivo *applications in animal models requiring high-titer LV vector stocks is challenging due to the lack of simple procedures allowing rapid processing of large volumes of LV vector-containing cell culture supernatants. The traditional ultracentrifugation-based methods are limited in terms of their capacity to handle large volumes, thus making this procedure extremely tedious. Filtration approaches such as diafiltration are well suited for processing large volumes of vector supernatants. However, they are difficult to implement in a standard laboratory setting. Thus, there is an emerging need for simple and less laborious procedures that result in a rapid reduction of the volume of the cell culture supernatant to be processed without the need for a centrifugation step.

One problem with the centrifugation and filtration-based methods outlined above is that cell-derived components are concentrated along with the vector particles. These have the potential to cause immune and inflammatory responses [15]. For example, concentrated VSV-G-pseudotyped LV vector preparations were shown to be contaminated with tubovesicular structures of cellular origin which carried nucleic acids, including the plasmid DNAs that were used to generate the LV vector stocks. DNA carried by these tubovesicular structures acted as a stimulus for innate antiviral responses, triggering Toll-like receptor 9 and inducing alpha/beta interferon production [16]. Thus, additional steps including chromatography-based methods such as anion exchange chromatography are needed in order to reduce host cell and cell culture-derived contaminants from LV vector preparations. Methods based on anion exchange column chromatography of LV vectors pseudotyped with VSV-G [17,18] or the baculovirus GP64 glycoprotein were previously described [19]. However, the yields and purity of the LV vector stocks obtained in this way were not reported.

In an attempt to simplify the production and concentration of LV vectors and to make this approach more reproducible and cost-effective for preclinical studies in animals, we have worked out a facile LV vector production system based on HYPERFlasks. We also implemented a straightforward concentration procedure based on Mustang Q anion exchange membrane chromatography. Mustang Q anion exchange-based chromatography protocols for concentrating/purifying LV vectors were previously reported [4,20]. Such vector preparations displayed reduced toxicity compared to vectors concentrated using ultracentrifugation [20], as well as enhanced purity [4].

## Results and discussion

### Simplified production of lentiviral vectors using HYPERFlasks

With a view toward improving high-titer LV vector production for preclinical studies in animals, we tested the usefulness of HYPERFlask vessels that have a total growth area of 1720 cm^2^, corresponding to ten standard T175 flasks. HYPERFlasks consist of ten interconnected growth surfaces each containing a membrane pretreated to allow improved cell adherence. The membrane is gas permeable, allowing exchange of oxygen and carbon dioxide through the base of the membrane, resulting in gas exposure to a large surface area within the flask [21].

To test the usefulness of HYPERFlasks for LV vector production involving calcium phosphate-mediated transfection [10], we compared the titers of LV vector stocks prepared side-by-side using either ten 150-cm^2 ^dishes or a single HYPERFlask. The data shown in Table 1 represent the results of three independent productions each for the 150-cm^2 ^dish system and the HYPERFlask system. These data indicate that the titers of unconcentrated LV vectors produced using HYPERFlasks were up to 2.3 × 10^8 ^TU/ml while the titers of LV vectors produced in 150-cm^2 ^dishes were lower, up to 6.9 × 10^7 ^TU/ml. The total yields were up to 1.2 × 10^10 ^TU from ten 150-cm^2 ^dishes and up to 1.3 × 10^11 ^TU for the HYPERFlask vessels. This corresponds to a productivity of up to 8 × 10^6 ^TU per cm^2 ^for the 150-cm^2 ^dishes. For the HYPERFlask, the productivity was about 10-fold higher, up to 0.75 × 10^8 ^TU per cm^2^. The higher productivity observed with HYPERFlasks may be related to better gas exchange during LV vector production. Overall, the 293T cells used for production appeared healthier and there was less cell debris from transfections carried out in HYPERFlaks compared to transfections carried out in 150-cm^2 ^dishes (data not shown). We expect the HYPERFlask production protocol described here to be compatible with other DNA transfection formats such as polyethylenimine-mediated transfection [22].

**Table 1:** Lentiviral vector production using 150-cm^2 ^dishes or HYPERFlasks

Production vessels	TU/ml	Total TU
150-cm^2 ^dishes	5.6 ± 1.3 × 10^7^	9.5 ± 2.1 × 10^9^
HYPERFlask	2.0 ± 0.3 × 10^8^	1.1 ± 0.16 × 10^11^

Ten 150-cm^2 ^dishes (total volume: 170 ml) and one HYPERFlask vessel (volume: 550 ml) were used for LV vector production. Productions were performed side-by-side (n = 3). Vector titers were determined using HOS cells. Data are expressed as the mean ± standard deviation (SD).

### Simplified concentration of lentiviral vectors using Mustang Q Acrodiscs

The concentration and purification of LV vector stocks on a large scale presents a significant bottleneck and methods allowing rapid processing of large volumes of LV vector-containing supernatants are needed. Chromatography-based methods appear to be particularly attractive in this regard [2]. For example, methods to concentrate/purify LV vectors based on anion exchange or affinity chromatography have been established [17-19]. Recently, Segura et al. [7] described an approach involving heparin affinity chromatography to concentrate LV vector particles directly from clarified supernatants. During this step, a recovery of 53% of infectious LV particles was achieved while 94% of the impurities were removed. This strategy may ultimately result in vector stocks of improved purity, increased infectivity and reduced toxicity. However, these approaches are cumbersome and difficult to implement in a standard laboratory setting.

We have recently described a facile membrane-based chromatography approach involving Mustang Q anion exchange chromatography cartridges (Acrodiscs) to purify adenoviral vectors [23]. Mustang Q membranes resulted in high recoveries of infectious viral particles and allowed the processing of adenoviral vector particles from lysates in a fraction of the time required using the traditional cesium chloride-based ultracentrifugation method. Mustang Q membranes include a matrix that has a high dynamic binding capacity for adenoviral vectors and is capable of withstanding high flow rates. Other benefits of Mustang Q membrane chromatography cartridges include higher peak resolution at faster flow rates as compared to traditional ion exchange resins. Also, they can easily be adapted to bench-scale work and they are syringe-adaptable. Finally, there is no need for an HPLC setup or a column packing step. In work with LV vectors carried out by us [20,24] and by others [4], Mustang Q-based cartridges were used to concentrate/purify LV vectors. While these initial studies were encouraging, the capacity of Mustang Q membranes for crude LV vector supernatants was rather low [24], possibly caused by cell-derived proteins, serum proteins, or contaminating nucleic acids that bound to Mustang Q membranes during vector capturing.

**Figure 1:**
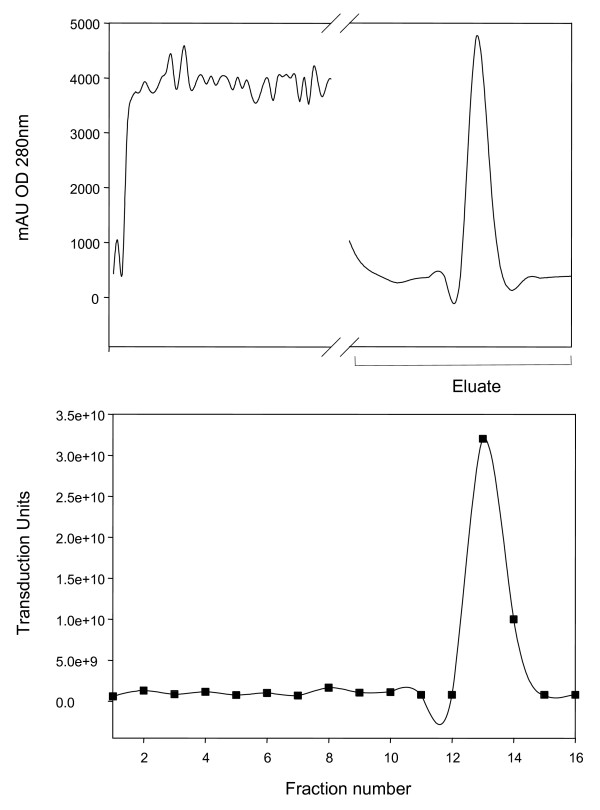
**Concentration of LV vectors by ion exchange chromatography using Mustang Q Acrodiscs**. A HYPERFlask vessel-derived LV vector-containing supernatant (500 ml) was adjusted to 25 mM Tris-HCl, pH 8.0, 0.3 M NaCl and loaded onto a Mustang Q Acrodisc (bed volume 0.18 ml) at a flow rate of 10 ml/min for 8 min using an AKTA purifier HPLC (Amersham Pharmacia) and Unicorn 4.0 Software (Amersham Pharmacia). The Acrodisc's membrane was washed with 2 ml of 25 mM Tris-HCl, pH 8.0, 0.3 M NaCl and LV vectors were eluted with a 10-ml gradient ranging from 0.3 to 1.5 M NaCl in 25 mM Tris-HCl, pH 8.0 and 1-ml fractions were collected. Flow through, wash, and eluate fractions were collected using a FRAC 950 collector (Amersham Pharmacia) and the OD at 280 nm was recorded (top panel); mAU = milli-absorbance units. Eluate fractions were immediately processed to analyze vector TU (bottom panel). The total TU for each fraction are presented.

To determine the binding capacity of Mustang Q membranes for LV vectors produced in HYPERFlask vessels compared to 150-cm^2 ^dishes, a capture study was conducted. Figure 1 shows a typical elution profile of HYPERFlask-produced LV vectors following capturing onto a 0.18-ml Mustang Q Acrodisc and elution using a gradient ranging from 0.3 to 1.5 M NaCl. TU were determined by FACS analysis of transduced HOS cells. The results presented in Table 2 show that the capacity of Mustang Q Acrodiscs at 10% breakthrough for LV vector supernatants produced in 150-cm^2 ^dishes was up to 3.9 × 10^9 ^TU per ml of membrane while the capacity of Mustang Q membranes at 10% breakthrough for HYPERFlask vessel-derived LV vector samples was up to 9.6 × 10^10 ^TU per ml of membrane. This is consistent with the observation that LV vector supernatants produced using HYPERFlasks contained fewer cellular proteins and nucleic acids compared to supernatants produced in 150-cm^2 ^dishes (Table 3) that may have interfered with the binding of the LV vector particles. To remove excess NaCl, pooled Mustang Q fractions were subjected to a buffer exchange using Sepharose CL-4B. Table 4 summarizes the recovery of LV vectors following processing of 500-ml aliquots of HYPERFlask vessel-derived supernatants. Up to 4.7 × 10^10 ^TU were obtained after Mustang Q chromatography and up to 5.3 × 10^10 ^TU after subsequent buffer exchange using Sepharose CL-4B (Table 4).

**Table 2:** Capacity of Mustang Q Acrodiscs for crude lentiviral vector stocks

Production vessels	Input (TU)	Unbound (TU)	Bound (TU)	Recovered (TU)	Yield	Capacity of Acrodisc (TU/ml)
150-cm^2 ^dishes	1.2 ± 0.5 × 10^9^	6.4 ± 5.1 × 10^8^	5.5 ± 1.6 × 10^8^	3.2 ± 0.8 × 10^8^	28.9 ± 5.4%	3.0 ± 0.9 × 10^9^
HYPERFlask	1.6 ± 0.1 × 10^10^	2.8 ± 6.4 × 10^8^	1.6 ± 1.6 × 10^10^	1.2 ± 0.1 × 10^10^	76.0 ± 0.70%	≥8.7 ± 0.9 × 10^10^

Mustang Q Acrodiscs were challenged with 75-ml aliquots of vector-containing supernatants. The crude vector supernatants were filtered using a 0.45 μm filter prior to anion exchange chromatography on Mustang Q Acrodiscs. Input corresponds to the total number of TU loaded. Unbound corresponds the total number of TU found in the flow-through. Bound represents input TU minus unbound TU. Recovered corresponds to the total number of TU following elution. Capacity corresponds to the total number of TU bound per ml of the Mustang Q membrane. Since the HYPERFlask-derived samples did not result in greater than 10% breakthrough, the upper limit of the capacity for these samples is not known. Titers were determined by FACS using HOS cells. Data from three independent experiments involving 150-cm^2 ^dish-derived and HYPERFlask vessel-derived vector supernatants are shown. The data are expressed as mean ± SD.

**Table 3:** Protein and DNA concentrations for crude lentiviral vector stocks

Production vessels	Protein (mg/ml)	DNA (μg/ml)
150-cm^2 ^dishes	4.35 ± 0.25	11.17 ± 1.6
HYPERFlask	3.03 ± 0.07	7.22 ± 0.32

Protein and DNA concentrations of 150-cm^2 ^dish-derived and HYPERFlask vessel-derived vector supernatants were determined using a Qubit assay kit (Invitrogen). Productions were performed side-by-side (n = 3). Data are expressed as mean ± SD.

**Table 4:** Recovery of lentiviral vectors following Mustang Q anion exchange chromatography and buffer exchange

Input (TU)	Unbound (TU)	Bound (TU)	Recovered following Mustang Q chromatography (TU)	Recovered following buffer exchange (TU)
7.3 ± 2.0 × 10^10^	7.4 ± 2.3 × 10^9^	6.5 ± 1.8 × 10^10^	4.1 ± 0.6 × 10^10^	4.5 ± 0.8 × 10^10^

Mustang Q Acrodiscs were challenged with 500-ml aliquots of HYPERFlask vessel-derived vector supernatants. Buffer exchange was carried out by Sepharose CL-4B size exclusion chromatography as outlined under Materials and Methods. Titers were determined by FACS using HOS cells. Data from three independent productions are shown. Data are expressed as mean ± SD.

Finally, we wanted to compare the titers and total yields of our concentrated LV vectors with those reported by other groups. To do this, we carried out a side-by-side titer comparison of a HYPERFlask-derived LV vector stock concentrated by Mustang Q anion exchange chromatography using HOS cells, 293T cells and HeLa cells. Table 5 shows that the titers of fresh (unfrozen) vector stocks were similar in HOS cells and 293T cells and slightly lower in Hela cells. Upon freezing and thawing of Mustang Q-concentrated vector stocks there was a 40% drop in vector titers on average (data not shown).

**Table 5:** Comparison of lentiviral vector titers using different cell lines

Cell line	HOS	293T	HeLa
Titer	3.1 ± 0.3 × 10^10 ^TU/ml	2.0 ± 0.3 × 10^10 ^TU/ml	9.0 ± 3.4 × 10^9 ^TU/ml

Freshly concentrated LV vector stocks were diluted and aliquots were used to transduce HOS, 293T and HeLa cells as outlined under Materials and Methods. Titers were determined by FACS (n = 3). Data are expressed as mean ± SD.

Overall, the titers obtained using the HYPERFlask system were as high or higher compared to those obtained using 150-cm^2 ^dishes and ultracentrifugation-based concentration approaches [3]. The yield (total TU) from a single HYPERFlask (cell growth area: 1720 cm^2^) was above 10^11 ^TU (Table 1). This is about two orders of magnitude higher than the yields reported by Karolewski et al. [5] who used a cell factory system (total cell growth area: 6320 cm^2^), and at least one order of magnitude higher than the yields reported by Geraerts et al. 2005 [6] who also used Cell Factories.

## Conclusion

In conclusion, we present here a simple protocol for LV vector production and concentration involving HYPERFlasks in conjunction with Mustang Q anion exchange membrane cartridges. These protocols are easy to implement in a standard laboratory setting and allow high-titer LV vector production without the need for multiple tissue culture dishes or flasks or ultracentrifugation-based concentration procedures.

## Methods

### Cell lines

293T cells (CRL-11268), human osteosarcoma (HOS) cells (CRL-1543) and HeLa cells (CCL-2) were obtained from the American Type Culture Collection (ATCC) and propagated in DMEM (high glucose), 10% fetal bovine serum (FBS, Invitrogen-GIBCO), 1% GlutaMAX (Invitrogen), 1% Pen/Strep (Invitrogen).

### Production of lentiviral vectors using 150-cm^2 ^dishes

LV vectors were generated by calcium phosphate-mediated transfection of 293T cells as described [10,25], with modifications. 293T cells were plated in 150-cm^2 ^dishes at a density of 8 × 10^6 ^cells per dish in 25 ml DMEM medium supplemented with 10% FBS, 1% Pen/Strep, 0.3% HyQ CelPro-LPS (HyClone). Twenty four h later, chloroquine (Sigma-Aldrich) was added to the medium at a final concentration of 25 μM. LV vector, packaging (helper), and envelope plasmid DNAs were combined in 3 ml of 0.25 M CaCl_2 _and then mixed with 3 ml of 2 × HEPES-buffered saline (2 × HBS) [26] using gentle vortexing. The DNA/CaCl_2_/HBS mixture was then added to medium. The amount of plasmid DNA used per dish was 21 μg of the pNL-EGFP/CMV/WPREΔU3 LV vector plasmid DNA [20] (Addgene plasmid 17579), 14 μg of the pCD/NL-BH*ΔΔΔ packaging plasmid DNA [27] (Addgene plasmid 17531) and 7 μg of the VSV-G-encoding pLTR-G plasmid DNA [12] (Addgene plasmid 17532). The medium was removed 16 h later and replaced with 17 ml of fresh DMEM/10% FBS/1% GlutaMAX per plate. Forty eight h later, the vector-containing medium was collected and spun at 500 × g for 5 min, filtered through a 0.45-μm pore size filter (Corning) and stored at -80°C.

### Production of lentiviral vectors using HYPERFlask vessels

293T cells (2 × 10^8 ^cells) were seeded into a HYPERFlask Cell Culture Vessel (Corning) in 550 ml of DMEM medium supplemented with 10% FBS, 1% GlutaMAX, 1% Pen/Strep, 0.3% HyQ CelPro-LPS. Twenty four h later, the medium was removed from the HYPERFlask vessel. Sixty ml of the medium were discarded, and chloroquine was added to the remaining medium at a final concentration of 25 μM. LV vector, packaging, and VSV-G plasmid DNAs were combined in 30 ml 0.25 M CaCl_2 _and mixed with 30 ml of 2 × HBS using gentle vortexing. The plasmid DNA/CaCl_2_/HBS mixture was combined with the remaining medium and transferred back into the HYPERFlask vessel. The total amount of plasmid DNA used was 210 μg of the pNL-EGFP/CMV/WPREΔU3 LV vector plasmid, 140 μg of the pCD/NL-BH*ΔΔΔ packaging plasmid, and 70 μg of the pLTR-G plasmid. The medium was removed 16 h later and replaced with 550 ml of fresh DMEM/10% FBS/1% GlutaMAX. Forty eight h later, the vector-containing medium was collected and filtered through a 0.45-μm pore size filter and stored at -80°C. Protein and DNA concentrations in vector supernatants were determined using a Qubit kit (Invitrogen) as recommended by the manufacturer.

### Determination of binding capacity of Mustang Q Acrodiscs for lentiviral vectors prepared using 150-cm^2 ^dishes or HYPERFlask vessels

To determine the binding capacity of Mustang Q Acrodiscs (PALL) for LV vectors, 75-ml aliquots of 150-cm^2 ^tissue culture dish-derived or HYPERFlask-derived vector supernatants were adjusted to 25 mM Tris-HCl, pH 8.0, 0.3 M NaCl and loaded onto a Mustang Q Acrodisc (bed volume 0.18 ml) at a flow rate of 10 ml/min for 8 min, using an AKTA purifier HPLC system and Unicorn 4.0 Software (Amersham Pharmacia). After loading, the Acrodisc's membrane was washed with 2 ml of 25 mM Tris-HCl, pH 8.0, 0.3 M NaCl. LV vectors were eluted with a 10 ml gradient ranging from 0.3 to 1.5 M NaCl in 25 mM Tris-HCl, pH 8.0. Flow through, wash, and eluate fractions were collected using a FRAC 950 collector (Amersham Pharmacia) and analyzed for vector TU.

### Concentration of lentiviral vectors using Mustang Q anion exchange chromatography

For concentrating LV vectors prepared using HYPERFlask vessels, 500-ml aliquots of vector-containing supernatants were adjusted to 25 mM Tris-HCl, pH 8.0, 0.6 M NaCl and loaded onto a Mustang Q Acrodisc (bed volume 0.18 ml) attached to an AKTA purifier HPLC system using the system's pump at a flow rate of 10 ml/min for 50 min. The Acrodisc's membrane was washed with 2 ml of 25 mM Tris-HCl, pH 8.0, 0.6 M NaCl, and vector particles were eluted with a step gradient ranging from 0.3 to 1.5 M NaCl in 25 mM Tris-HCl, pH 8.0. The flow through, wash, and eluted fractions (1 ml per fraction) were collected using a FRAC 950 collector and immediately analyzed for TU. To desalt the vector samples, the eluted fractions showing the highest TU levels were pooled, loaded onto a 2.0-ml Sepharose CL-4B column (Amersham Pharmacia) equilibrated with Tris-HCl, pH 7.4, and 150 mM NaCl. The column was spun at 500 × g for 1 min and the excluded volume was immediately analyzed for TU. The vectors were stored at -80°C.

### Vector titration using FACS

To determine vector titers (TU), HOS cells, HeLa cells or 293T cells were plated in 6-well plates at a density of 5 × 10^4 ^cells per well in DMEM high glucose medium supplemented with 10% FBS, 1% GlutaMAX, 1% Pen/Strep. The next day, the medium was removed and 0.5 ml of medium containing 8 μg/ml polybrene was added. Aliquots of concentrated LV vector preparations diluted 1:500 or of unconcentrated preparations were added. After incubation overnight, the medium containing the vector samples was removed and 2 ml of fresh medium was added to each well. Seventy two h after vector addition, cells were processed for FACS analysis [28].

## Authors' contributions

RK, SP, MM and JR conceived and designed the experiments. SP and RK performed the LV vector production and RK and MM optimized the Mustang Q anion exchange membrane chromatography steps. JR and RK wrote the manuscript. All authors read and approved the final manuscript.
